# Nuclear quiescence and histone hyper-acetylation jointly improve protamine-mediated nuclear remodeling in sheep fibroblasts

**DOI:** 10.1371/journal.pone.0193954

**Published:** 2018-03-15

**Authors:** Luca Palazzese, Marta Czernik, Domenico Iuso, Paola Toschi, Pasqualino Loi

**Affiliations:** Faculty of Veterinary Medicine, University of Teramo, Teramo, Italy; Peking University Third Hospital, CHINA

## Abstract

Recently we have demonstrated the possibility to replace histones with protamine, through the heterologous expression of human protamine 1 (hPrm1) gene in sheep fibroblasts. Here we have optimized protaminization of somatic nucleus by adjusting the best concentration and exposure time to trichostatin A (TSA) in serum-starved fibroblasts (nuclear quiescence), before expressing Prm1 gene. To stop cell proliferation, we starved cells in 0.5% FBS in MEM (“starved”—ST group), whereas in the Control group (CTR) the cells were cultured in 10% FBS in MEM. To find the most effective TSA concentration, we treated the cells with increasing concentrations of TSA in MEM + 10% FBS. Our results show that combination of cell culture conditions in 50 nM TSA, is more effective in terminating cell proliferation than ST and CTR groups (respectively 8%, 17.8% and 90.2% p<0.0001). Moreover, nuclear quiescence marker genes expression (*Dicer1*, *Smarca 2*, *Ezh1* and *Ddx39*) confirmed that our culture conditions kept the cells in a nuclear quiescent state. Finally, ST and 50 nM TSA jointly increased the number of spermatid-like cell (39.4%) at higher rate compared to 25 nM TSA (20.4%, p<0.05) and 100 nM TSA (13.7%, p<0.05). To conclude, we have demonstrated that nuclear quiescence in ST cells and the open nuclear structure conferred by TSA resulted in an improved Prm1-mediated conversion of somatic nuclei into spermatid-like structures. This finding might improve nuclear reprogramming of somatic cells following nuclear transfer.

## Introduction

The spermatozoon is the perfect device for nuclear transfer. Its compact genome is easily transported to the fertilization site, and the reprogramming machinery of the oocyte, evolutionarily adapted to its DNA packaging structure, reveals its intrinsic totipotency. Spermatogenesis is a stepwise process that includes several biological events leading to the progressive destabilization of the nucleosome that is propaedeutic to the protamine-driven toroid DNA conformation Luca, I am not sure I understand what you try to say here. Propaedeutic is British spelling so if use, need to spell propaedeutic. I would not use this or toroid as these are very uncommon words. Maybe replace with introductory and convoluted, respectively? [1]. Nucleosome destabilization starts with the synthesis and assembly of testis-specific histone variants [2–4], followed by their post translational modification, namely acetylation [5], and then by bromodomain protein DNA binding [6]; Transition nuclear protein and protamine assembly completes the process [3]. The idea of turning the nuclear structure of somatic cells into a spermatozoa-like structure is thus appealing and might be a worthy strategy to improve nuclear reprogramming of somatic cells when used for somatic cell nuclear transfer (SCNT). We have recently moved along this path, demonstrating that the heterologous expression of human Protamine1 (hPrm1) in somatic cells (fibroblasts) re-shapes the interphase nuclei into spermatid-like structure [7]. Fibroblasts “protaminization” was effective but limited, with only 30% of them acquiring a spermatid nuclear shape. In this work, we have radically improved these outcomes by mimicking the physiological nuclear remodeling occurring in elongated spermatids. Nuclear quiescence and histone hyper-acetylation of post-meiotic round spermatids are crucial features for successful nuclear remodeling. In this work, we have further refined the conditions to induce nuclear quiescence and histone hyper-acetylation in sheep adult fibroblasts (SAFs), in order to maximize their nuclear protaminization. Suboptimal culture conditions, such as high cell density [8], and removal of serum [9] drive cells into nuclear quiescence. However, the metabolic hold occurring in starved cells causes a reduction of transfection efficiency [10], jeopardizing the possibility for improved reprogramming. For this reason, we decided to exploit a moderate serum starvation with a subtle addition of trichostatin A (TSA). TSA, the most used histone deacetylase inhibitor (HDAI), also induces H3-4 acetylation [11, 12] leading to a proliferation arrest at G0/G1 at nanomolar concentration [13] in mouse embryonic cells [14], fibroblast [15] and in several kinds of cancer cells [16, 17].

Here, we report a synergy between serum starvation and TSA in inducing nuclear quiescence, leading also a histone hyper-acetylation. Resulting from these is an improved efficiency of somatic chromatin protaminization in SAF transfected with mouse Protamine 1 (mPrm1).

## Materials and methods

### Ethics statement

Animal work (skin biopsy) has been approved by the Italian Ministry of Health, upon the presentation of the research description prepared by the ethics committee of the Istituto Zooprofilattico Sperimentale di Teramo (Prot. 944F0.1 del 04/11/2016). The number of the authorization granted by the Italian Ministry of Health is n° 200/2017-PR.

### Cell culture

**Fig 1:**
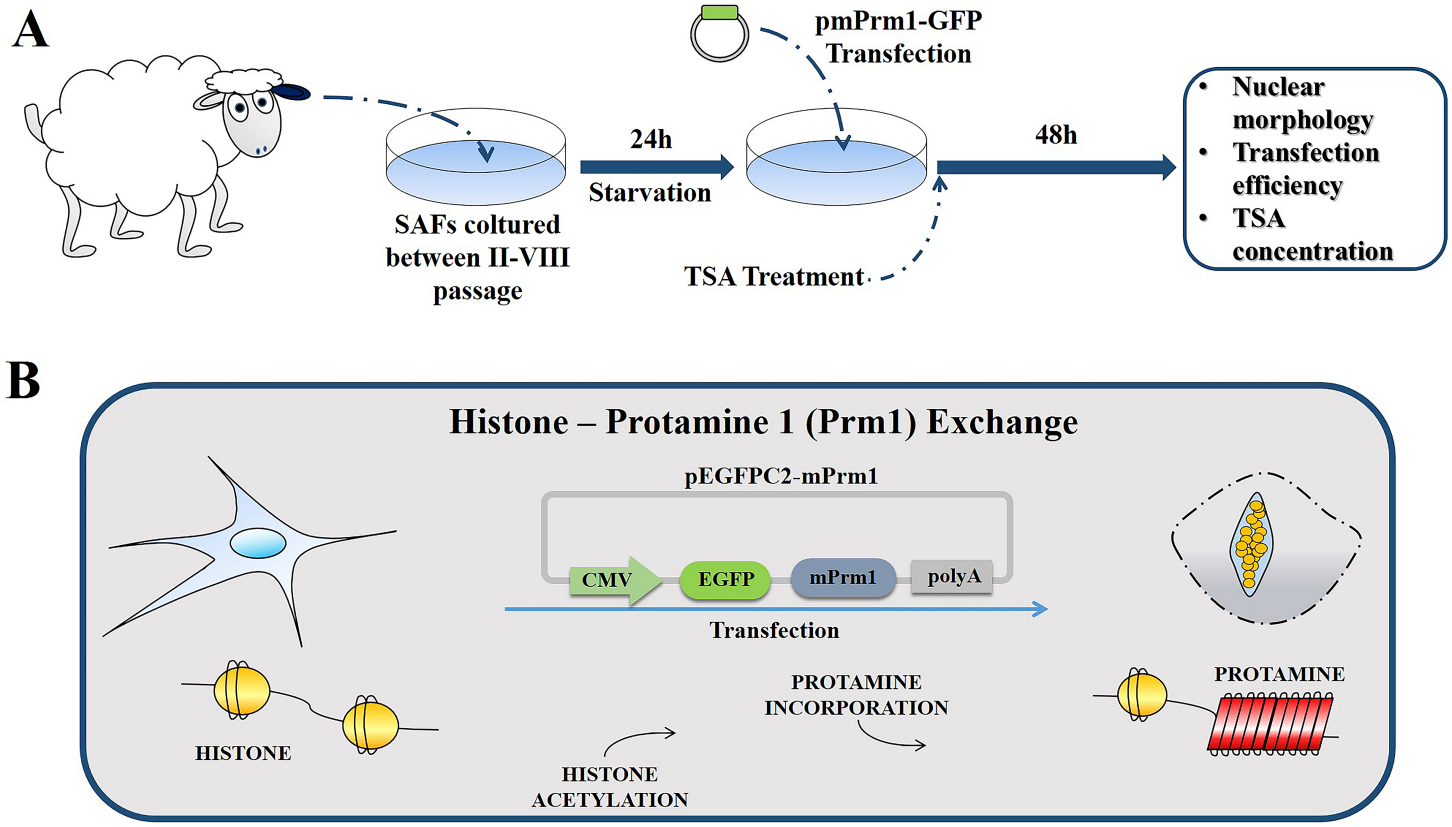
Schematic representation of experimental design. (A) Sheep adult fibroblasts (SAFs) were isolated from ear biopsies and cultured between two and eight passages. Twenty-four hours before transfection, SAFs were cultured with 0.5% FBS and various concentrations of trichostatin A (TSA). SAFs were transfected with pEGFPC2-mPrm1, and nuclear remodeling and transfection efficiency were evaluated 48 h after transfection. (B) SAFs transfected with pEGFPC2-mPrm1 replaced somatic histone with mouse Prm1 and compacted the nucleus in a spermatid-like structure.

SAF were derived from ear biopsy of three female Sarda breed sheep (2 years old). SAFs (between second and eighth passage) were cultured in DMEM (GIBCO) containing 2 nM glutamine, 3.7 g/L NaHCO_3_, and 0.5% gentamicin supplemented with 10% Fetal Bovine Serum (FBS), (Control group—CTR). To induce a quiescent sate (starvation), SAFs were cultured for 24h in DMEM supplemented with 0.5% FBS (ST-group) (Fig 1A).

### Plasmid construction

Mouse sperm *Prm1* (GenBank: NP_038665.1) was amplified from a 50-day-old mouse testis cDNA library with appropriate primers and cloned into a pEGFPC2 vector (Evrogen). The identity of the cloned cDNA and its in-frame cloning C-terminal to GFP was verified by sequencing.

### Transfection and TSA treatment

Transfection was performed as previously reported [18] (Fig 1B). Briefly, SAF at 80% confluence were transfected with 3 μg of mPrm1-GFP by lipofectamine 2000 (Invitrogen) according to the manufacturer’s instructions with small modification. From 4h post-transfection to end of protaminization (48h after transfection), medium was changed to DMEM containing different concentration of histone deacetylase inhibitor (HDAI) tricostatin A (TSA): 25 nM (25 TSA), 50 nM (50 TSA), or 100 nM (100 TSA). Selection of TSA concentrations was done on the basis of published reports by us [18] and others [15]. To evaluate transfection efficiency and mPrm1 presence in the nucleus, cells were stained with H33342 and GFP+ cells were then counted in relation to total cells count. At least 200 nuclei were analyzed for each group for statistical soundness.

To evaluate chromatin remodeling by mPrm1-GFP we proceeded in the following two approaches: First, SAFs were grown on a 22 × 22 mm glass cover slip, and 24h after transfection were fixed with 1 mL of 4% Paraformaldehyde. DNA counterstaining was done with DAPI. SAFs were then mounted on slides with Fluoromount^™^ aqueous mounting medium (Sigma) and analyzed on a confocal microscope (Nikon Eclipse Ti-E); Second, since the fully compacted cells detach from the Petri dish surface and float in the medium, the medium with the cells was recovered and centrifuged (1500 rpm for 5 minutes) 48h after transfection and TSA exposure. The supernatant was discarded and the pellet was re-suspended and fixed in 1 mL of 4% of paraformaldehyde. DNA counterstaining was done by DAPI, and the slides were mounted as described above. Images were captured by confocal microscope (Nikon Eclipse Ti-E) and both analysis and three-dimensional reconstruction were developed by NIS-Elements Confocal software (Nikon).

### Cell proliferation assay

Cell proliferation assay was performed on SAFs cultured for 24 hours under CTR (MEM + 10% FBS), ST (MEM + 0.5% FBS) and 50 TSA (MEM + 10% FBS + 50 nM TSA) conditions and evaluated by an indirect immunocytochemistry assay for 5-bromo-2'-deoxyuridine (BrdU) detection, a thymidine analogous incorporated during S-phase. Briefly, SAFs of CTR, ST and 50 TSA groups were cultured with 100 μM BrdU for 6 hours before the end of culture, fixed in cold methanol for 20 minutes, and permeabilized at room temperature (RT) with 0.1% Triton-X-100 (in PBS) for 15 minutes. Next, cells were treated with 4N HCl at RT for 30 minutes and incubated with a primary antibody (mouse anti-BrdU, monoclonal antibody, B2531, Sigma) at a rate of 1:100 in blocking solution (0.1% bovine serum albumin (BSA) in PBS) at 4°C, overnight. Cells were then incubated with a secondary antibody (rabbit anti-mouse IgG-FITC polyclonal antibody, F9137, Sigma) at a rate of 1:500 in BS at RT for 2 hours and counterstained with 0.5 μg/mL propidium iodide (PI) at RT for 5 minutes. Between every passage, cells were washed twice with PBS at RT for 5 minutes. The number of proliferative cells was calculated for each group by calculating the ratio between the number of BrdU-positive cells and the total number of nuclei. At least 200 nuclei were analyzed for each group for statistical significance.

### Gene expression analysis

Since BrdU incorporation only shows cells in active DNA synthesis but not cells at other stages, we analyzed the expression of the main gene markers of nuclear quiescence according to a recent review on the topics [19]: *Dicer1*, *Smarca2*, *Ezh1* and *Ddx39*.

Total RNA from CTR, ST and 50 TSA—groups (5 replicates for each group) was extracted using an RNeasy Mini Kit (Quiagen, Milan, Italy) according to the manufacturer’s instruction. Samples were reverse-transcribed using GoScript^™^ reverse transcription system (Promega, Milan, Italy) and used for gene expression analysis employing specific 5’-3’ primer pairs designed to anneal at 56/58°C with amplification efficiency (E) range between 2.1 to 1.9 (S1 Table). Real Time PCR was carried out using SsoAdvanced Universal SYBR Green Supermix (Bio-Rad, Milan, Italy), according to the manufacturer’s instructions. Relative gene expression was calculated using the comparative threshold cycle method (ΔΔCT) with μTUBULIN and SDHA as housekeeping genes. Dissociation analysis was performed in every run to check the specificity of each amplification.

### H3 acetylation

H3 Acetylation was performed by immunostaining and Western immunoblotting as described below.

#### Acetyl-Histone H3 immunostaining

Acetylation of Histone H3 was performed on SAFs cultured for 24h under 0 nM TSA (0 TSA), 25 TSA, 50 TSA and 100 TSA conditions by indirect immunocytochemistry assay. Briefly, cells were fixed with 4% paraformaldehyde for 15 minutes at RT and washed with PBS. Blocking was done with blocking buffer (2% Triton-X-100 + 0.1% BSA in PBS) for 15 minutes. Next, cells were incubated with primary antibody (Rabbit Anti-Acetyl-Histone H3 polyclonal antibody, Millipore, cat. 06–599), diluted 1:100 in blocking buffer, for 4 hours at RT. Then, the cells were washed in PBS containing 2% Triton-X-100 and incubated with secondary antibody (Anti-Rabbit IgG (whole molecule)–FITC antibody produced in goats, Sigma, F0382) at dilution of 1:400 in PBS containing 2% Triton-X-100 for 1 hour at RT. Subsequently, cells were washed with PBS to remove the unbound secondary antibody. Finally, DNA counterstaining was performed with 0.5 μg/mL DAPI for 5 minutes. Images were acquired on confocal microscope (Nikon Eclipse Ti-E). Laser power for detection of acetyl-histone H3 was set at 1% for all readings.

#### Acetyl-Histone H3 Western immunoblotting

Total protein extracts were obtained from SAFs previously treated with 0, 25, 50 or 100 nM of TSA using radioimmunoprecipitation assay (RIPA) buffer. Then 50 μg of total extract were denatured by heating at 95°C for 5 min in 1% (v:v) sodium dodecyl sulphate (SDS), 1% (v:v) β -mercaptoethanol, 20% (v:v) glycerol in 50 mM Tris–HCl at pH 6.8. Samples were subjected to electrophoresis in 4–15% SDS polyacrylamide gels. After electrophoresis proteins were transferred to 0.2 μm PVDF membrane. Membrane were blocked in TBS-T [0.2% (v:v) Tween-20 in 20 mM Tris, 137 mM NaCl at pH 7.6] with 5% (w:v) skimmed milk for 1 hour, and then washed three times in TBS-T at room temperature (RT). Membranes were incubated with primary rabbit polyclonal anti-trimethyl-Histone H3 antibodies (Lys9) (Cat. #07–523, Millipore) and anti-Actin antibody (used as loading control) (sc-1615, Santa Cruz Biotechnology, Santa Cruz, USA). Both were diluted in 0.1% blocking solution at a ratio of 1:1000 and incubated at 4°C overnight. After three washes with TBS-T, membranes were incubated at RT with secondary antibodies at 1:1000 in 0.1% blocking solution for 1 h. After three washes in TBS-T, the final detection was performed by enhanced chemiluminescence using the Westar nC Ultra 2.0 Western blotting detection system (Cyangen, Bologna, Italy). Image acquisition was carried out using the ChemiDoc System (Bio-Rad, Milan, Italy).

### Statistical analysis

Data were analyzed using GraphPad Prism for Windows (Version 6.01, GraphPad Software, Inc, CA, USA). Statistical analyzes of transfection yields and cell proliferation were based on five replicates per experiment and were compared using the Fisher’s exact test. Data in gene expression analysis were reported as means ± standard error of the mean (SEM) and were analyzed using the non-parametric Mann-Whitney *U* test. The level of significance was set at *P* < 0.05.

## Results

### Inducing nuclear quiescence by culturing the fibroblasts with low serum concentration and trichostatin A

**Fig 2:**
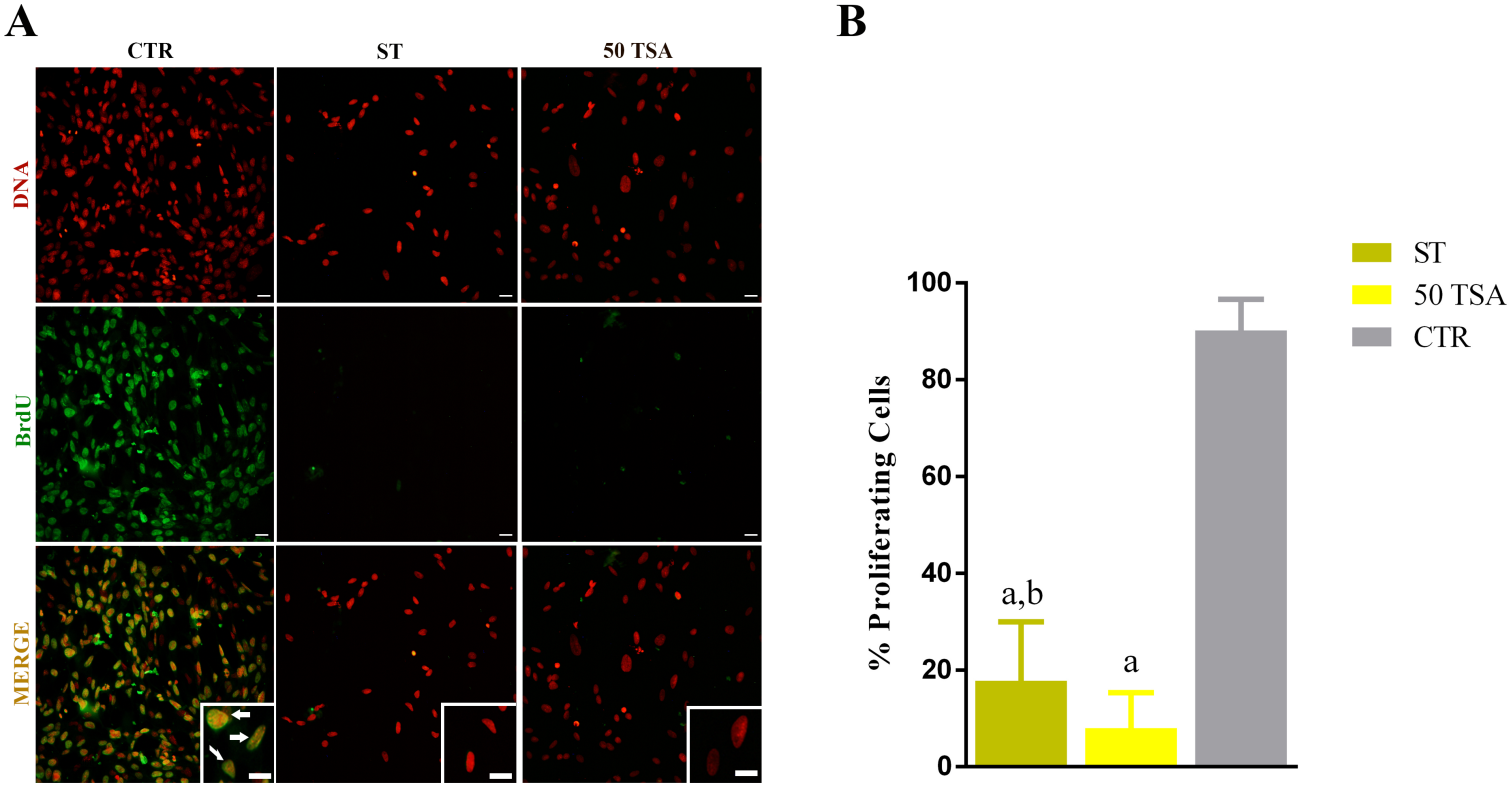
Cell proliferation assay. (A) BrdU immunocytochemistry assay. Left column: control group (CTR), centre: “starved” group (ST), right column: 50 trichostatin A group (TSA). Top row: DNA stain (propidium iodide); central row: BrdU immuno-detection; bottom row: merge. White arrows indicate cells in active proliferation. Scale bars represent: 20 μm. (B) Graphic represents percentage of proliferative cells. “a” mean value *P*<0.0001, ST and 50 TSA versus CTR; “b” mean value p<0.0001, ST versus 50 TSA.

In order to find the optimal nuclear quiescence induction still compatible with a satisfactory vector transfection, we took advantage of a combination of moderate serum starving (compatible with efficient transcription of the vector) and cell cycle blocking properties of Trichostatin A (TSA). BrdU incorporation assay demonstrated a near absence of proliferation in serum starved fibroblasts (ST; 17.8% of cells proliferating, n = 234) and TSA (50 TSA, 8% of cells proliferating n = 372) groups compared to the control group (CTR; 90.2% of cells proliferating n = 912; Fig 2A and 2B).

**Fig 3:**
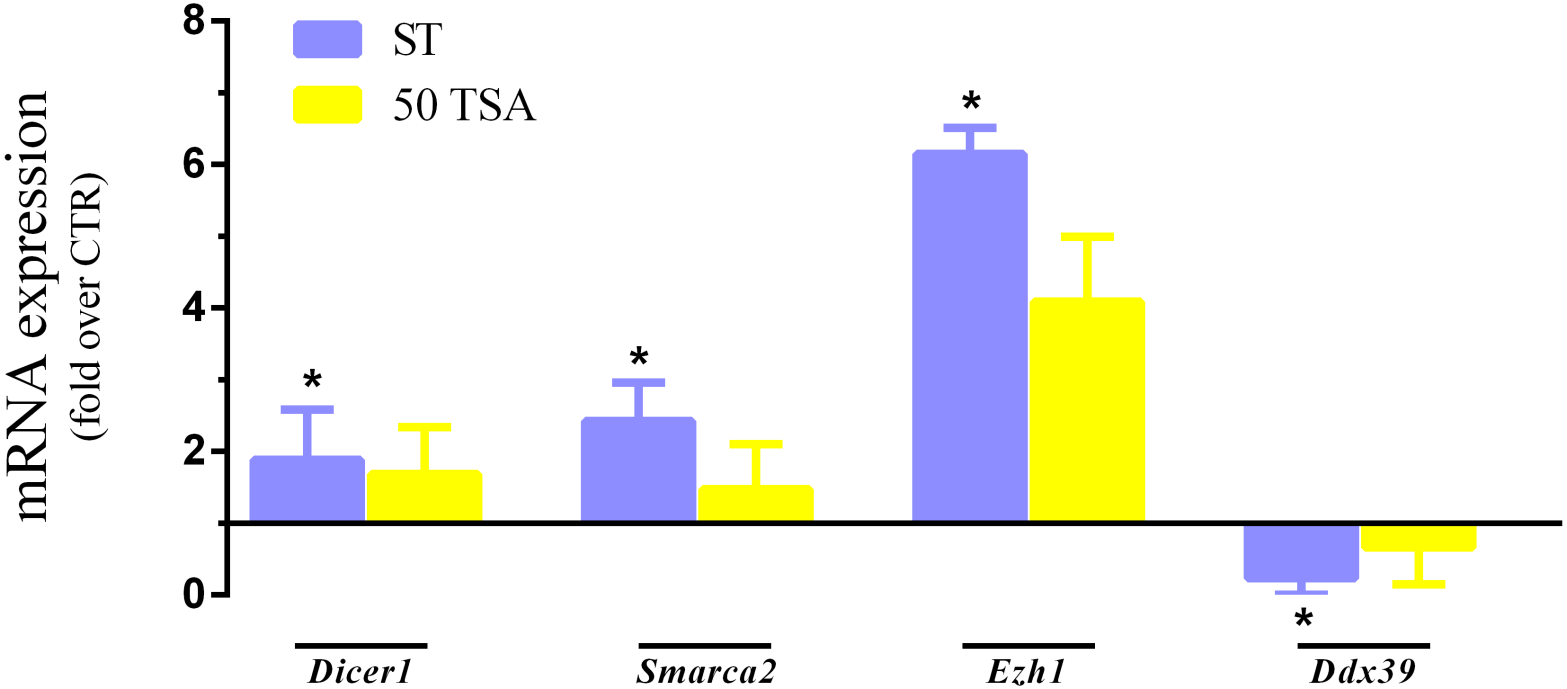
Gene expression analysis. * mean value. *P* < 0.05.

Nuclear quiescence markers *Dicer1*, *Smarca 2*, and *Ezh1* were highly expressed in ST and 50 TSA compared to the CTR group, with significant differences particularly between ST and CTR. On the other hand, ST and 50 TSA groups showed lower expression levels of *Ddx39* compared to the CTR group, again with a significant downregulation particularly between ST and CTR (Fig 3). Thus, serum starving/TSA combination resulted in a very efficient way to induce nuclear quiescence.

### Induction of genome hyper-acetylation by increasing concentration of histone deacetylase inhibitor trichostatin A

**Fig 4:**
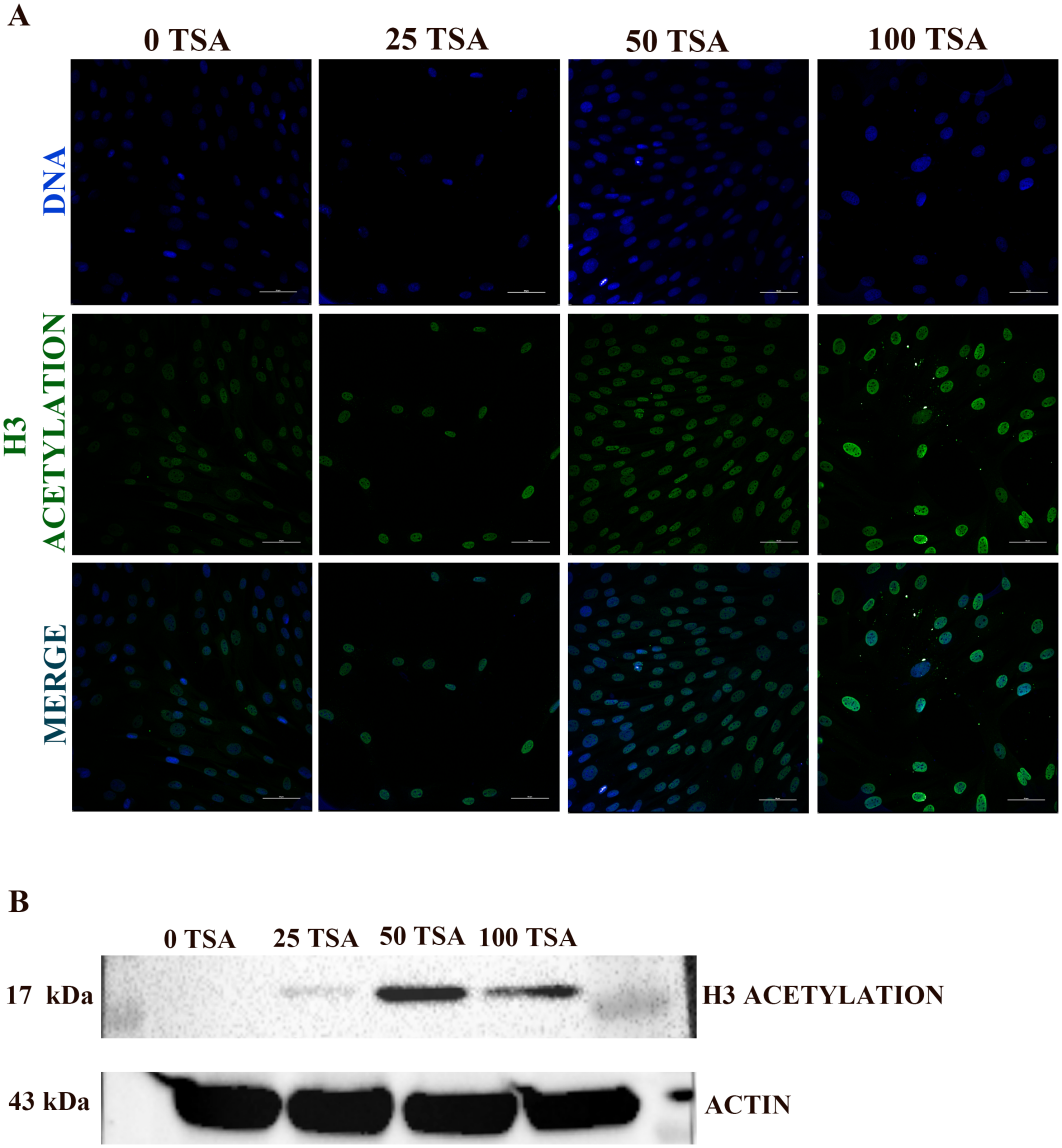
Acetyl-Histone H3. (A) Acetyl-Histone H3 Immunostaining. Left column: 0 TSA; second: 25 TSA; third: 50 TSA; fourth: 100 TSA. Top row: DNA stained with DAPI; centre row: Acetyl-Histone H3 immunofluorescence; bottom row: merge. Scale bars represent: 50 μm. (B) Acetyl-Histone H3 Western immunoblotting.

TSA is a common drug used to “open” the chromatin structure thanks to its histone hyper-acetylation effect. TSA however is toxic to the cells. It is therefore compulsory to carefully adjust the dosage, particularly in conditions such as ours, where the nuclei are later used for nuclear transfer as even minimal cell damage can affect future development of the cloned embryos. Sheep adult fibroblasts (SAFs) treated with 0, 25, 50 and 100 nM TSA showed histone H3 acetylation (Fig 4A and Table 1). As shown in Fig 4A, there is a positive relationship between TSA concentration and the presence of acetyl groups in histone H3. Moreover, Western immunoblotting showed a better histone H3 acetylation at TSA concentration of 50 nM (Fig 4B). Hence, this TSA concentration was used in the transfection essays.

**Table 1:** Data of Acetyl-Histone H3 immunostaining analysis by confocal microscopy.

SAMPLE	Laser Power; channel DAPI (%)	High Voltage; channel DAPI	Laser Power; channel FITC (%)	High Voltage; channel FITC
0 TSA	30	98	1	100
25 TSA	30	98	1	100
50 TSA	13.54	98	1	100
100 TSA	7.18	98	1	100

**Fig 5:**
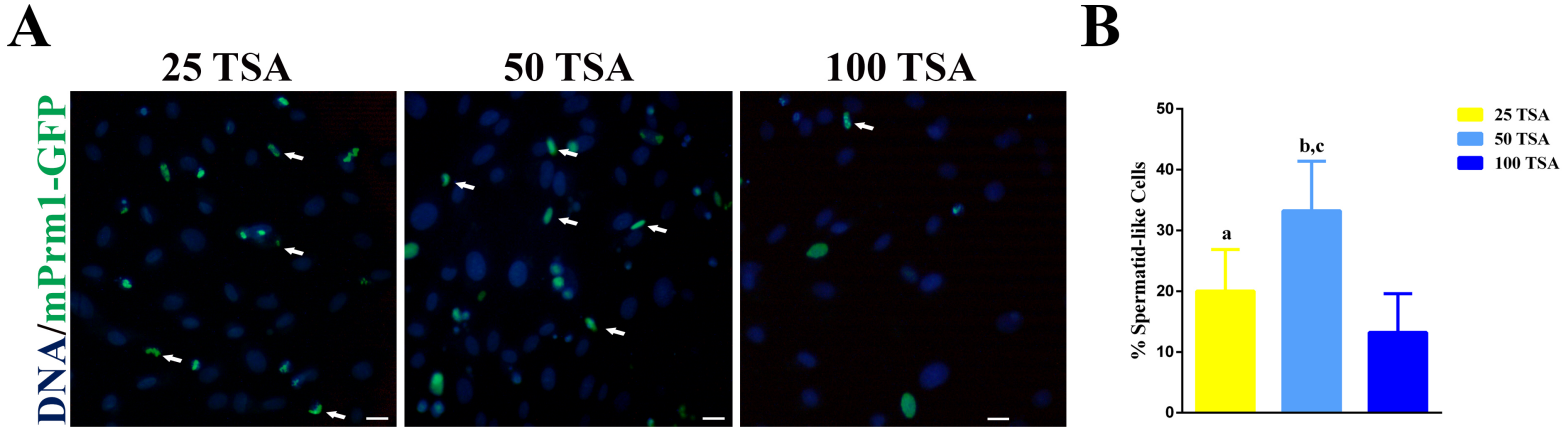
Number of spermatid-like cells at 48 h post-transfection. (A) Fluorescence images of TSA-treated cells. White arrows indicate spermatid-like cells. Scale bar represents 10 μm. (B) Graphic representation of spermatid-like cells at 48 h after transfection in relation of TSA concentration: “a” mean value *P* = 0.0314 25 TSA versus 100 TSA; “b” mean value *P* = 0.0059 50 TSA versus 25 TSA; “c” mean value *P*<0.0001 50 TSA versus 100 TSA.

The proportion of spermatid-like cells, assessed 48 h after transfection, was higher in the 50 TSA group, with a significant drop in the 100 TSA group (25 TSA; 20.4%, 50 TSA; 30.4%, 100 TSA; 13.7% n = 306) as shown in Fig 5.

### Evaluation of nuclear remodeling efficiency in transfected sheep fibroblasts following nuclear quiescence induction and genome hyper-acetylation

**Fig 6:**
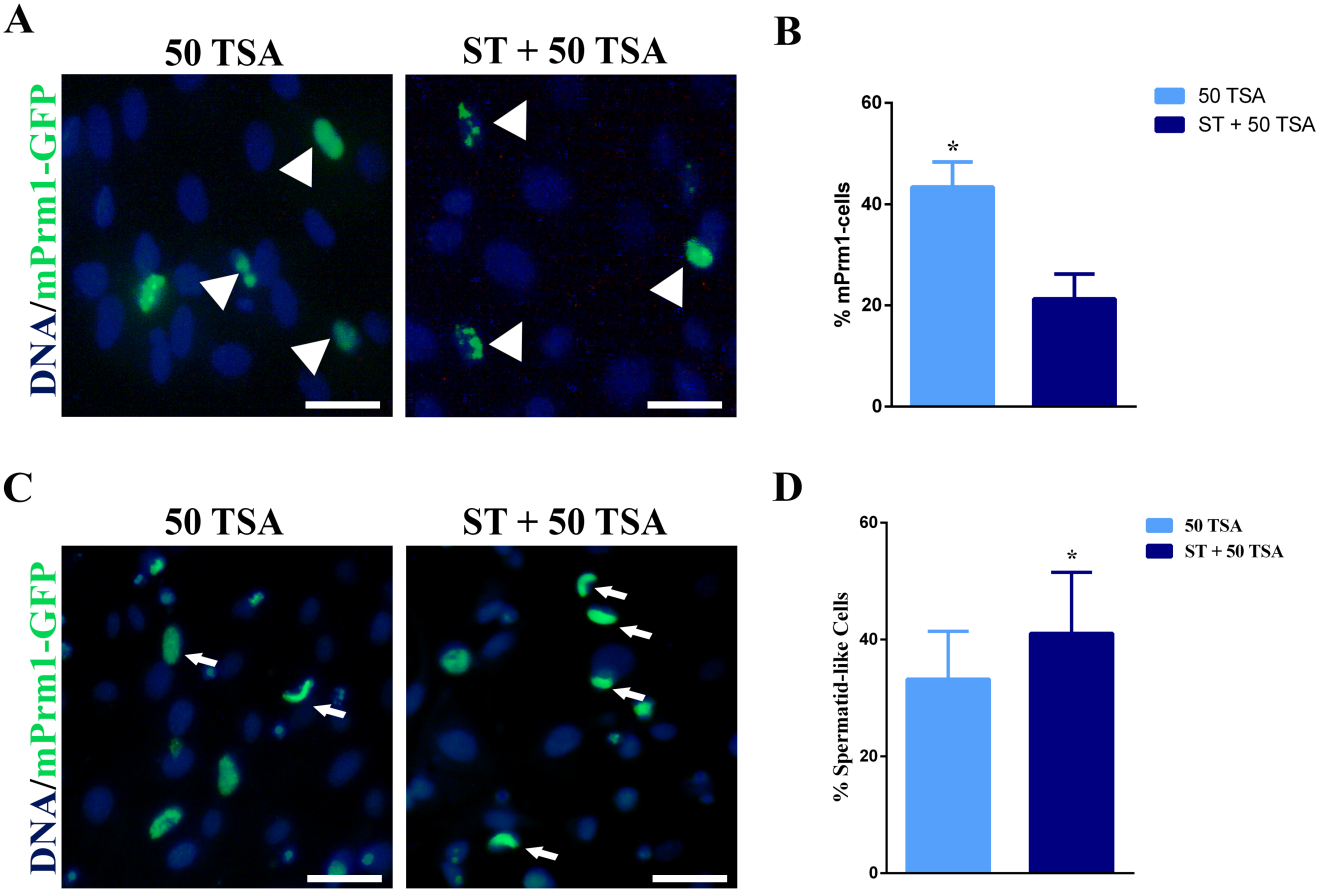
Number of mPrm1-transfected cells at 24 h after transfection and number of spermatid-like cells at 48 h post-transfection. (A) Fluorescence microscopy of 50 TSA and ST + 50 TSA group at 24 h post-transfection. The white arrowheads in the figure indicate mPrm1-transafected cells. (B) Graphic representation of the number of mPrm1-transfected cells at 24 h after transfection. * Mean value *P*<0.05. (C) Fluorescence microscopy of 50 TSA and ST + 50 TSA group at 48 h post-transfection. White arrows in the figure indicate spermatid-like cells. (D) Graphic representation of the number of spermatid-like cells at 48 h after transfection. * Mean value *P* = 0.0301. Scale bars represents: 20 μm for A and C.

The efficiency of SCNT is strictly dependent on nuclear remodeling of the transferred nuclei. We have hypothesized that induction of chromatin conformation similar to spermatid should enhance SCNT efficiency. Hence, in this experiment we have quantified the number of spermatid-like cells obtained following the different treatments tested prior to cells transfection with protamine 1 vector. Although transfection efficiency at 48h in ST + 50 TSA group was lower than in 50 TSA group (21.4% vs. 43.6%, respectively; Table 2), the former displayed higher number of spermatid-like cells (39.4%), with an extensive nuclear protaminization (51.4%) comparing to the latter (30.4% and 37%, respectively; Table 2 and Fig 6).

**Table 2:** Nuclear remodeling outcome at 24h-48 h after transfection.

Culture Condition	No. Cells	mPrm1-cells (%)	mPrm1 in entire nucleus (%)	Spermatid-like cells (%)
**0 TSA**	607	167 (27.5)	52 (31.1)	37 (22.2)
**ST**	844	33 (3.9)	17 (51.5)^a^	9 (27.3)
**50 TSA**	656	286 (43.6)^b^	125 (37.0)	87 (30.4)
**ST + 50 TSA**	594	127 (21.4)	73 (51.4)^c^^,^ ^d^	50 (39.4)^e^^,^ ^f^

^a)^ ST VS 0 TSA, mean value, p<0.05.

^b)^ 50 TSA VS ST + 50 TSA, mean value, p<0.05.

^c)^ ST + 50 TSA VS 0 TSA, mean value, p<0.05.

^d)^ ST + 50 TSA VS 50 TSA, mean value, p<0.05.

^e)^ ST + 50 TSA VS ST, mean value, p<0.05.

^f)^ ST + 50 TSA VS 50 TSA, mean value, p<0.05.

Therefore, data shows that ST + 50 TSA group is the most effecting in inducing and *in vitro* remodeling somatic cell nuclei into spermatid-like structure; thus, presumably, more easily reprogrammable by the oocyte following SCNT.

### Mouse Prm1 completely remodels sheep adult chromatin

**Fig 7:**
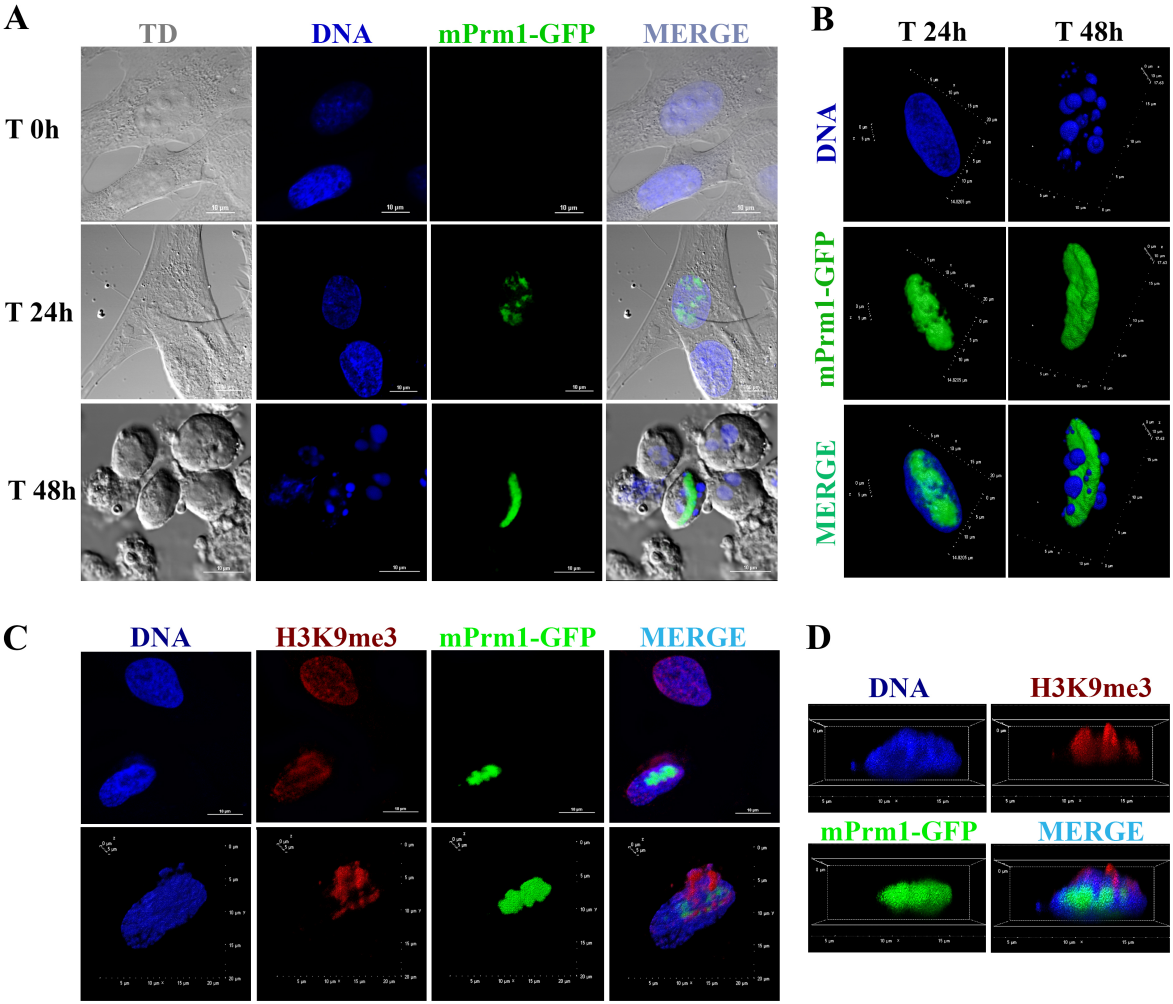
Nuclear protaminization and H3K9me3 immunoassaying. (A) Time dependent mPrm1-GFP incorporation. Upper Row: SAFs at time 0h; Middle row: SAFs at 24 h after transfection; Bottom row: SAFs 48 h after transfection. Left: Transmission DIC (TD); Second; DNA stained with DAPI; Third row: mPrm1-GFP signal; Fourth row: Merge. Scale bar: 10μm. (B) Three-dimensional (3D) reconstruction of protaminized nuclei using confocal microscopy. Left: SAF at 24 h after transfection; Right: SAF at 48 h post-transfection. In the top row: DNA stained with DAPI; Middle row: mPrm1-GFP signal; Bottom row: Merge. (C) H3K9me3 immunoassay 24 h post-transfection. Left: DNA stained with DAPI; Second: H3K9me3 immuno-detection; Third: mPrm1-GFP signal; Fourth; Merge; Top row: Two-dimensional image; Bottom row: 3D reconstruction. (D) Cropped 3D image reconstruction of H3K9me3 immunoassay 24 h post-transfection. Scale bars represent: 10 μm for A and C.

Sheep genomics and is far less advanced than in the mouse. As a consequence, while exhaustive research tools are available for the latter species, our model, the sheep, lags behind from this point of view. Here we wanted to test whether the easily available mouse Prm1 vector can remodel sheep somatic cells. After transfection, SAFs showed progressive nuclear compaction that was completed by 48 h. The dynamics of chromatin compaction was similar to what we have previously described [7]. The chromatin starts to compact 24 h post transfection, with a few foci of mPrm1-GFP. At 48h after transfection the cells nuclei acquire a spermatid-like structure (Fig 7A and 7B), detach from the Petri dish, and float in the medium. The nuclear remodeling induced by mPrm1-GFP also displaces the histone H3 trimethylation on lysine 9 (H3K9me3) (Fig 7C and 7D). Thus, mouse Prm1 vector successfully remodels sheep somatic cells.

## Discussion

Here we have demonstrated that association of serum starvation with TSA treatment increases the number of fully protaminized cells in mPrm1-transfected fibroblasts.

Starved cells are less prone to plasmid transfection [10]. Since transfection is a key step in our protaminization protocol, we decided to use a “mild” (1% instead of the canonical 0.5% serum deprivation capable to keep enough cell metabolism to allow an adequate vector uptake). TSA is a well-characterized genome hyper-acetylation agent that also induces a synchronous G0/G1 block owing to the up-regulation of p21, p27 and p53 proteins that collectively negatively interfere with bulk transcription [17].

Pulse exposure to BrdU and its immune-detection is the golden standard for the assessment of cell cycle block in cell culture [20]. In reality, BrdU incorporation assay documents only the presence or absence of DNA synthesis but does not indicate the entrance into nuclear quiescence. Given the novelty of our cell synchronization approach, we set up for a deeper evaluation of cell cycle in treated cells, using a recently characterized battery of genes unequivocally expressed in nuclear quiescence [19]. mRNA expression analysis indicated an up-regulation of *Dicer1*, *Smarca2*, *Ezh1* and a down-regulation of *Ddx39* in ST group, a profile typically found in quiescent cells [19]. Therefore, a synergistic effect between moderate serum starvation and moderated TSA treatment (50 nM) resulted in a robust cell cycle block at G0 in SAFs; synergy witnessed also in the increase of TSA-induced histone H3 acetylation (Fig 4).

The 50 TSA group displayed a higher number of mPrm1 expressing cell compared to ST + 50 TSA, probably because of the higher metabolism in the former group that might have positively affected transfection efficiency. However, ST + 50 TSA group showed a higher number of mPrm1 in whole nucleus and spermatid-like cells at 48 hours post-transfection (respectively: 51.4% vs. 37.0% and 39.4% vs. 30.4%, *P*<0.05 for both). Thus, the synergy between 50 TSA and serum starvation positively affects not only cell cycle blocking, but also nuclear remodeling in mPrm1-transfected cells. G0 stage is characterized by displacement of transcription factors from the DNA [21]; therefore, it might be that the combination of TSA-mediated genome hyper-acetylation and displacement of transcription factors in G0 have induced a high permissive state for mPrm1 deposition on the genome. To conclude, we have demonstrated that the combination of serum starvation and TSA treatment jointly induces nuclear quiescence in somatic cells, resulting, at the same time, in a better nuclear protaminization upon mPrm1 transfection.

These findings are of relevance for SCNT for several reasons. Cell cycle remains a central issue in SCNT. Even though there is some criticism on the effective role of G0 as a better condition for nuclear reprogramming, indeed G0/G1 remains the best cell cycle option for SCNT [22]. In addition to that, our finding indicates that G0 is also the best cell cycle stage for nuclei protaminization. These findings might be a worthy strategy for improving SCNT, even though *in vitro* nuclear transfer experiments and *in vivo* trials are required to have definitive answers.

Another potential application of our results might be found in biobanking for biodiversity preservation. We and others have shown that genomes can be preserved in a dry state, while maintaining the potentiality to produce normal embryos upon SCNT [23, 24]. However, drying negatively affects DNA integrity [25], and thus possibly compromises *in vivo* development of such cloned embryos. Spermatozoa have been the first eukaryotic cells to be stored in a dry form and still able to generate offspring following intracytoplasmic sperm injection (ICSI) [26–29]. Chromatin conformation in spermatozoa is highly favorable for dehydration, as its final nuclear organization is resistant to physical stressors, including radiation, or drying [30]. Protaminization of somatic cells, as achieved under the conditions herein described, might confer a better desiccation tolerance to somatic cells, thus facilitating drying of cell lines collected from endangered species (and otherwise) for biodiversity preservation, to be eventually used through SCNT.

## Supporting information

S1 Table Primer list of gene used for gene expression analysis. Primers pairs used for gene expression analysis with reference number (National Center for Biotechnology Information, NCBI), primer forward and revers sequence, number of base pair and annealing temperature.
(DOCX)
