# Supplementary material for: Nuclear quiescence and histone hyper-acetylation jointly improve protamine-mediated nuclear remodeling in sheep fibroblasts
Source: PLoS One. 2018 Mar 15;13(3):e0193954. doi: 10.1371/journal.pone.0193954 (PMC5854339; doi:10.1371/journal.pone.0193954)
Supplement: S1 Table — Primers pairs used for gene expression analysis with reference number (National Center for Biotechnology Information, NCBI), primer forward and revers sequence, number of base pair and annealing temperature. (DOCX) [file pone.0193954.s001.docx]

**Supporting Information**

**S1**

The following table (S1 Table) shows the primer list used for gene expression analysis to assess the correct nuclear quiescent.

**S1 Table. Primer list of gene used for gene expression analysis.**

| **GENE** | **PRIMER SEQUENCE** | **BASE PAIR** | **Annealing T.** |
| --- | --- | --- | --- |
| ***DICER1***  (XM_004017979.3) | **Fw** AAG GAA GCT GGC AAA CAA GA  **Rw** AAA AGC AAC CAC CAA GTT GC | 228 | 58˚C |
| ***SMARCA2***  (XM_004004345.3) | **Fw** TTG GCC AGC AAA ATG AGG TC  **Rw** TTT CCT CTT CGT GCT CCA GT | 192 | 58˚C |
| ***EZH1***  (XM_012186127.2) | **Fw** AGT CTC TGC ACT CCT TCC AC  **Rw** CAT GGC GTA CTC CTT TGC TC | 182 | 58˚C |
| ***DDX39***  (XM_004008473.3) | **Fw** GGG ACG TGC AGG AAA TCT TC  **Rw** ACT CCA ACA CAT CCA GGA GG | 234 | 60˚C |
| ***SDHA***  (DQ386895.1) | **Fw** AGC AGA AGA AGC CGT TTG AG  **Rw** TGC GTC TGC TTC AAA GTC CT | 121 | 58˚C |
| ***µTUBULIN***  (NM_001009284.2) | **Fw** CCA TCC AGC GTA TTC CAG AG  **Rw** GCG TGG GAC AGA AGG TAG AA | 202 | 56˚C |

Primers pairs used for gene expression analysis with reference number (National Center for Biotechnology Information, NCBI), primer forward and revers sequence, number of base pair and annealing temperature.
